# BiMba: using Vision Mamba to predict protein sites that bind other proteins

**DOI:** 10.1093/bioinformatics/btag243

**Published:** 2026-07-07

**Authors:** Azam Shirali, Parshatd Govindasamy, Vitalii Stebliankin, Jimeng Shi, Kalai Mathee, Giri Narasimhan

**Affiliations:** Bioinformatics Research Group (BioRG), Knight Foundation School of Computing and Information Sciences, Florida International University, FL 33199, United States; Bioinformatics Research Group (BioRG), Knight Foundation School of Computing and Information Sciences, Florida International University, FL 33199, United States; Euleris Inc., Miami, FL 33140, United States; Bioinformatics Research Group (BioRG), Knight Foundation School of Computing and Information Sciences, Florida International University, FL 33199, United States; Euleris Inc., Miami, FL 33140, United States; Bioinformatics Research Group (BioRG), Knight Foundation School of Computing and Information Sciences, Florida International University, FL 33199, United States; Euleris Inc., Miami, FL 33140, United States; Biomolecular Sciences Institute, Florida International University, Miami, FL 33199, United States

## Abstract

**Motivation:**

Identifying protein binding sites in protein–protein complexes is a central challenge in structural biology. Binding sites, consisting of groups of residues, govern how proteins recognize, and interact with protein partners. Thus, identifying them is essential for understanding biological function and guiding the design of effective biomolecules and even drug molecules. Despite major progress in computational approaches, their performance remains limited because most models underrepresent the combined influence of surface properties and residue-level information, leaving room for improvement. Recent advances in state-space models and vision-based deep learning offer an opportunity to address these limitations by efficiently modeling long-range spatial dependencies on protein surfaces. Here, we introduce BiMba (protein *Bi*nding site prediction using Vision *M*am*ba*), a state-space–driven deep learning framework that leverages the efficient long-range modeling capability of the Vision Mamba architecture to learn from three-dimensional (3D) protein surfaces represented as two-dimensional (2D) geometric or physicochemical grids.

**Results:**

BiMba integrates complementary sources of information, capturing geometric and physicochemical determinants of molecular recognition as surface patches, encoded as 2D images, along with residue-level descriptors, yielding a unified representation that couples spatial topology with biochemical context. BiMba demonstrates competitive performance across diverse and specialized benchmark datasets, often outperforming existing state-of-the-art methods. In addition, BiMba incorporates perturbation-based and gradient-based interpretability analyses by extracting hidden attentions from Mamba layers, enabling visualization of feature relevance and biologically meaningful residue clusters. Overall, our findings establish state-space models as efficient, interpretable, and scalable architectures for molecular surface learning, advancing the application of deep learning in structural bioinformatics.

**Availability and implementation:**

The BiMba source code, training, test, and benchmark datasets are available at https://github.com/Azam-Shi/BiMba.

## 1 Introduction

Proteins carry out many of their biological functions through interactions with other proteins. These interactions occur on a protein’s surface at specific protein binding sites, identified often by the interacting residues. Predicting these interactions and identifying binding sites remains one of the central and most challenging problems in structural bioinformatics. Proteins also interact with small-molecule ligands such as drug molecules, but their binding sites occur in localized pockets and their prediction requires capturing broader and more complex surface patterns driven by shape and physicochemical complementarity (Gainza *et al.* 2025, Yuan *et al.* 2025). Binding sites are crucial for understanding a protein’s function and for guiding the design of effective therapeutic strategies. Knowing the exact location of a protein’s binding site enables us to recognize the key residues involved and to design methods to strengthen or disrupt these interactions for medical or biotechnological applications (Kuzmanov and Emili 2013, Konc and Janežič 2022). In this work, we focus exclusively on protein–protein binding.

Recent advances in deep learning (DL) have substantially improved protein–protein interaction prediction, particularly through cofolding, graph-based, and surface-based approaches (Gainza *et al.* 2025, Shirali *et al.* 2025a, Yuan *et al.* 2025). These methods represent and encode protein structural and biochemical information in different ways. Representations include point clouds (dMaSIF; Sverrisson *et al.* 2021), three-dimensional (3D) voxel grids or distance/orientation maps (BiteNet; Kozlovskii and Popov 2020), sequence information converted to two-dimensional (2D) profiles (DeepDISOBind; Zhang *et al.* 2022), and graphs that capture spatial proximity (GraphRBF; Zhang *et al.* 2024). More citations can be found in a recent survey (Shirali *et al.* 2025a). However, most chemical interactions occur on protein surfaces, making surface properties the most critical features. HCGNet (Lin *et al.* 2024) integrates complementary atomic-level and residue-level properties. MaSIF-site (Gainza *et al.* 2020) maps geometric and physicochemical features onto a triangulated mesh (Sanner *et al.* 1996), followed by geometric DL. This method focuses on point-level features, neglecting residue-level properties.

As reviewed (Shirali *et al.* 2025a), complementary residue-level descriptors, such as polarity, residue depth, and amide plane orientation link point-level and residue-level characteristics, but have been largely overlooked. Recently, PIsToN (Stebliankin *et al.* 2023, Shirali *et al.* 2025b) demonstrated that converting MaSIF’s surface patches into structured 2D images and using powerful vision models result in improved predictive performance (Shirali *et al.* 2025a). Here, we introduce BiMba (protein *Bi*nding site prediction using a Vision *M*am*ba*), a DL framework for binding-site prediction that converts surface features to 2D images, but after integrating both surface- and residue-level features during training, leading to a more comprehensive and biologically informed understanding of protein binding sites.

Contributions: Here, we make the following novel contributions:

- Comprehensive feature integration. BiMba integrates physicochemical, surface-level and residue-level features, enabling better modeling of molecular interactions.
- Residuewise point selection strategy. By using a residue-wise point selection approach BiMba assigns each surface vertex to a residue, guaranteeing fair contribution from all residues, and learning contextual relationships between points and residues.
- Vision-Mamba network. By leveraging the state-space models and vision-based DL in Vision Mamba (ViM), BiMba efficiently captures global dependencies to improve binding site predictions over competing approaches. The novelty here is in integrating rich surface-based representations with complementary residue-level features in a unified framework.
- Explainability. BiMba provides interpretable predictions by analyzing hidden attention dynamics within Mamba. Visualizations of feature importance and gradients offer biological insight into predictions.
- Comprehensive testing. Rigorous benchmarking on large datasets shows that BiMba performs well against competitors.

## 2 Materials and methods

### 2.1 Data preprocessing and model design

Extending an approach introduced in MaSIF-site (Gainza *et al.* 2020) and improved by PIsToN (Stebliankin *et al.* 2023) for extracting protein surface features, we projected the surface patches into 2D image-like representations, including new features and preserving their spatial continuity and local topology. Binding sites occur not due to individual surface-exposed residues alone, but from specific spatial arrangements of physicochemical features across a region (Gainza *et al.* 2025). BiMba captures this by using images of features from gridded regions of the protein surface.

To prepare the data, we began with the 3D structures of protein complexes, each consisting of two interacting proteins. The complexes were first separated into their individual chains to allow independent surface analysis of each partner. Each protein was then preprocessed by removing heteroatoms, water molecules, and alternate atom positions to obtain clean and consistent inputs. The surface of each protein was triangulated and rescaled to a granularity of 1 Å.

The surface was then subdivided into overlapping patches centered on surface vertices within a fixed geodesic radius, capturing both geometric and local physicochemical information. For each surface point, we computed shape index, curvature, electrostatic potential, hydropathy, and hydrogen-bonding propensity features using MaSIF-site data preparation modules. As the last step of the preprocessing phase, we then extended this process by mapping the point-level features of each patch onto a circular 2D grid to preserve their spatial relationships. This mapping to grids encodes the geometric and chemical landscape of the protein surface, providing rich and suitable inputs for our vision-based model to learn patterns associated with binding sites.

### 2.2 Network architecture

Recent breakthroughs in computational structural biology with AlphaFold3 (Abramson *et al.* 2024) have demonstrated the remarkable power of core attention layer in transformer-based architectures (Vaswani *et al.* 2017) in modeling complex biomolecular systems and their interactions. However, emerging studies in natural language processing (NLP) and computer vision suggest that state space models (SSMs) (Gu *et al.* 2020), such as Mamba (Gu and Dao 2024) for sequential data and ViM (Zhu *et al.* 2024) for visual data (Shirali *et al*. 2026), can achieve representational capacity that is comparable to transformers while offering greater efficiency in capturing long-range dependencies and better scalability across sequence or image lengths. Motivated by these advances, we developed BiMba, a Vision Mamba-based framework for protein binding site prediction that leverages the efficiency of SSMs to learn spatial and biochemical patterns from structured protein surface representations.

**Figure 1:**
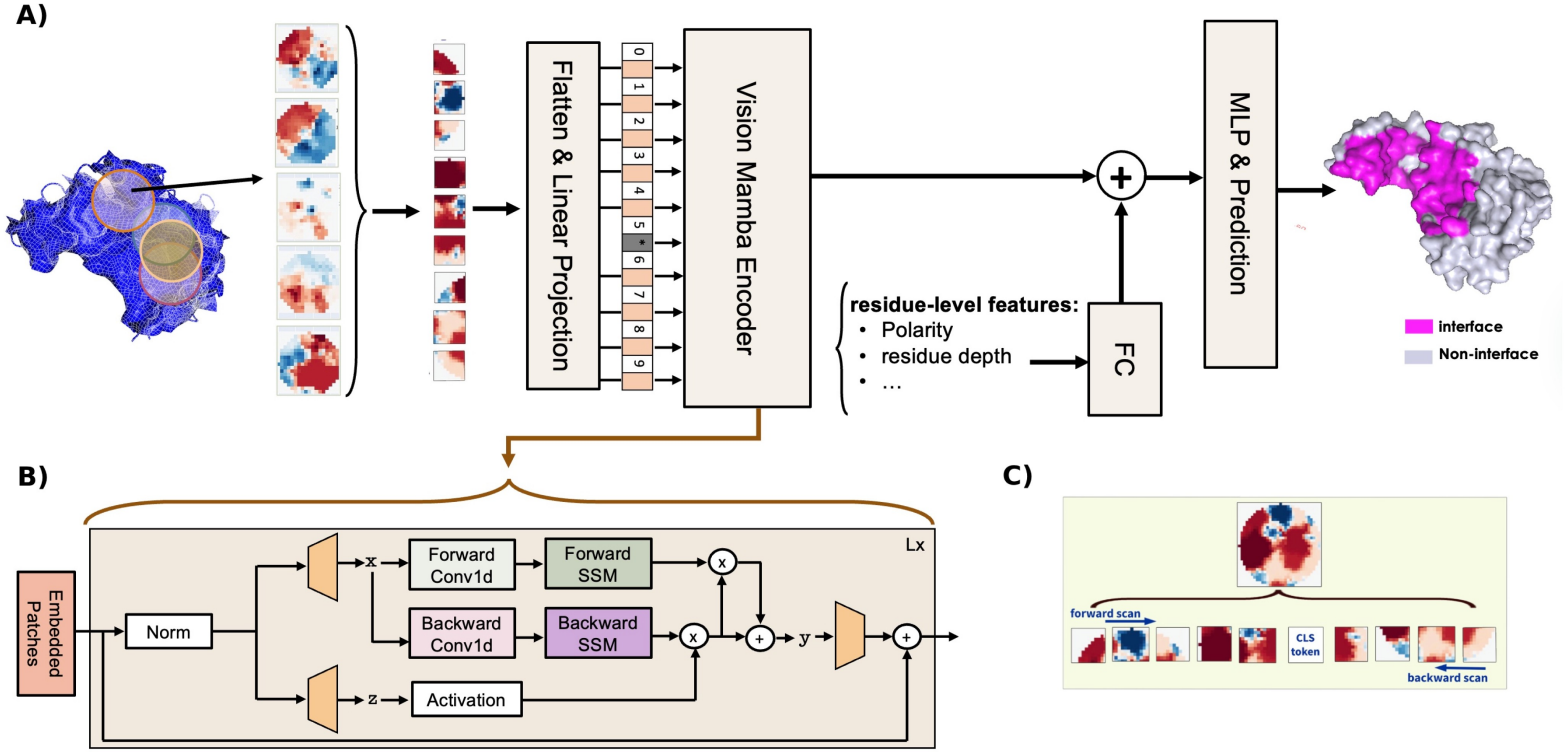
Overview of the BiMba framework and its ViM-based architecture. (A) The overall BiMba pipeline, where protein surface patches are projected into 2D grids, embedded using the ViM encoder, and concatenated with residue-level features for binding-site prediction. (B) The internal structure of the ViM encoder, illustrating bidirectional selective state-space modeling with forward and backward scanning. (C) Illustration of the bidirectional processing of surface patches and CLS token within the ViM encoder.

In BiMba, the generated surface grids are processed through the ViM architecture to extract compact latent embeddings that encode geometric and physicochemical patterns of the protein surface (Fig. 1). The resulting embeddings were then concatenated with complementary residue-level descriptors, including residue depth (Afsar Minhas *et al.* 2014), protrusion index (Kahraman *et al.* 2007), coordinate number (Afsar Minhas *et al.* 2014), secondary structure (Touw *et al.* 2015), and amide plane orientation (Morehead *et al.* 2023) to capture local geometric information, as well as polarity (Yan *et al.* 2008), relative solvent accessibility (Rost and Sander 1994), and the one-letter residue name (residue type) to encode global context (see Section C for details, available as supplementary data at *Bioinformatics* online). Together, these integrated features enrich the representation by linking point-level surface properties with intrinsic residue-level properties. This integration enhances the model’s sensitivity to subtle binding patterns and residue-specific contributions that are overlooked by MaSIF-site, which is a geometric DL model trained purely on point-level surface representations. To determine the optimal architecture, we performed an ablation study in which each component of the model was added or removed in isolation. The results clearly indicate that the best-performing model is obtained when all residue-level descriptors are combined with the 2D surface maps, providing a principled basis for model selection.

### 2.3 Training process

For the training process, we used the dataset introduced in MaSIF-site (Gainza *et al.* 2020), which contains 3314 PDB protein structures. The dataset includes 2958 proteins for training and 356 for testing. Following Gainza *et al.* (2020), we further split 10% of the training set for validation based on the pairwise TM-score matrix (see Gainza *et al.* 2020 for details). To address the inherent class imbalance in binding-site prediction—where noninterface residues greatly outnumber interface residues—we employed focal loss (Lin *et al.* 2017). Only about 10%–15% of the residues in a typical protein are located within binding sites, making this imbalance a significant challenge for learning. The focal loss function downweights easy negative samples and emphasizes harder, misclassified positive examples, thereby improving the model’s ability to learn discriminative features for interface residues. Each training batch contained a single protein, allowing the model to process all surface patches and residue-level features of that protein together.

### 2.4 Surface point selection strategy

For each protein, we computed a triangulated molecular surface and, for every vertex, identified its nearest atom using a *K*-nearest neighbor search; the vertex was then assigned to the residue containing that atom, thereby establishing a residue–surface correspondence. Afterward, all surface points belonging to each residue were collected. However, since proteins—even of moderate size—can contain an extremely large number of surface vertices, we applied a controlled downsampling strategy to ensure computational efficiency while allowing every residue to contribute during training. In other words, all interface-associated points were retained, while a predefined fraction of noninterface points was randomly selected based on a predefined threshold (see Section B for details, available as supplementary data at *Bioinformatics* online). This training approach guarantees that every residue contributes to the training process, preventing the omission of residues that might otherwise be ignored under random sampling strategies such as that used in MaSIF-site. By enforcing residuewise selection, the model learns from all residues, not only those at the interface, capturing how nearby noninterface residues shape the interfacial context through topology, electrostatics, and steric effects. This helps the model build a more complete and context-aware representation of binding behavior across proteins.

During inference, a deterministic, biochemically guided selection strategy was applied to identify representative surface points per residue. The selection was based on a hierarchical prioritization of atomic groups: reflecting the natural order of their contribution to molecular recognition—polar and charged atoms defining specificity, aromatic groups providing stacking and hydrophobic packing, backbone atoms contributing to secondary interactions, and aliphatic groups forming the stabilizing core (see Section B for details, available as supplementary data at *Bioinformatics* online). This hierarchy mirrors established biochemical observations that polar and aromatic residues govern interaction specificity, whereas hydrophobic residues primarily stabilize the interface structure.

### 2.5 Software and hardware

The distances between atomic coordinates were computed using KDTree from the SciPy package v1.1.0 and FLANN (fast library for approximate nearest neighbors) was employed for efficient *K*-nearest neighbor searches during surface-to-atom mapping. The neural networks were implemented in PyTorch v1.10. Residue-level features were computed using the feature-generation tools described in Morehead *et al.* (2023). Training and testing of the DL models were performed on a GeForce GTX 1080 Ti (8 GB GPU) system with 256 GB RAM and a 28-core Intel Xeon CPU E5-2650, as well as on the IRCC computing resources at FIU.

## 3 Results

The predictive power of BiMba was compared with several state-of-the-art methods including GraphRBF, and other baselines including SPPIDER (Porollo and Meller 2007), GraphPPIS (Yuan *et al.* 2022), MaSIF-site (Gainza *et al.* 2020), and ScanNet (Tubiana *et al.* 2022). The comparison metrics include accuracy (ACC), recall (Rec), precision (Pre), F1-score (F1), area under the receiver operating characteristic curve (AUROC), and area under the precision–recall curve (AUPRC).

From a geometric perspective, the ability of a method to accurately recover the physical interface area of a protein reflects its capacity to identify the true interaction region. To assess this, we propose a novel geometry-aware, area-weighted AUROC, where the ROC curve is constructed by weighting each residue according to its physical area, allowing us to quantify the interface region correctly recovered and missed.

### 3.1 P-250-test set

The first evaluation dataset used in this study is the P-250-test set introduced by Zhang *et al.* (2024) in the GraphRBF work. This set contains 250 nonredundant and diverse protein structures with varying sizes and lengths derived from the Protein–Protein Docking Benchmark (DBD 5.5) and Dockground databases.

Results shown in Table 1 include performance data for SPPIDER, GraphPPIS, MaSIF-site, and ScanNet reported in Zhang *et al.* (2024). As these results were directly adopted from the original study, AUC (area) could not be computed for this test set.

**Table 1:** Performance of different methods across different datasets.

Method	ACC	Rec	Pre	F1	AUC	AUC (area)	PRC
P-250-test set							
SPPIDER	0.81	0.42	0.19	0.27	0.73	–	0.18
GraphPPIS	0.84	0.43	0.23	0.30	0.75	–	0.21
MaSIF-site	0.75	0.42	0.17	0.26	0.73	–	0.18
ScanNet	0.85	0.42	0.29	0.34	0.80	–	0.26
GraphRBF	0.89	0.43	**0.37**	0.39	**0.83**	–	**0.37**
BiMba	**0.90**	**0.44**	0.35	**0.40**	0.78	–	0.29
MaSIF-test set							
MaSIF-site	0.75	0.34	0.23	0.27	0.85	0.78	0.28
dMaSIF	0.79	0.36	0.27	0.31	0.87	0.80	0.33
HCGNet	0.79	0.37	0.34	0.33	**0.89**	0.83	0.38
GraphRBF	0.78	0.23	**0.41**	0.24	0.70	0.67	0.35
BiMba	**0.83**	**0.56**	0.36	**0.44**	0.88	**0.88**	**0.41**
PINDER-S set							
MaSIF-site	0.71	0.26	0.21	0.22	0.65	0.69	0.28
dMaSIF	0.70	**0.56**	0.27	0.38	**0.70**	0.72	0.32
HCGNet	0.80	0.30	0.33	0.31	0.67	0.74	0.29
GraphRBF	0.83	0.10	0.34	0.16	0.63	0.68	0.25
BiMba	**0.87**	0.48	**0.35**	**0.42**	0.69	**0.81**	**0.35**

Bold values indicate the highest values under corresponding metrics. “–” indicates values not available.

Our model outperforms the competition in accuracy, recall, and F1 score, while GraphRBF is marginally better in precision, AUC, and PRC. BiMba achieves excellent discriminatory performance while using new features and architectural design. BiMba’s higher sensitivity to true binding residues suggests that it captures more biologically relevant binding regions at the cost of precision. Although BiMba was trained on bound structures, it remains competitive on this benchmark—drawn from docking datasets where unbound conformations are common—indicating that the model generalizes beyond its training conformational regime. While GraphRBF may have shown higher precision, AUC, and PRC values for this dataset, its advantage disappeared when we experimented later with a second dataset that included many unseen proteins.

### 3.2 MaSIF-test set

The MaSIF-test set (Gainza *et al.* 2020) covers a wide range of interaction types and protein sizes obtained from well-known, multiple sources, including PRISM, ZDock, PDBBind, SAbDab (see Gainza *et al.* 2020 for details). Results in Table 1 show that BiMba outperformed the competing models with the highest ACC, Rec, F1-score, and PRC values, demonstrating a superior ability to detect true interface residues while minimizing false positives. Although HCGNet reached a slightly higher AUC, BiMba achieved the best area-weighted AUC, indicating that it more effectively recovered the true physical interface region on the protein surface.

### 3.3 PINDER-S set

Recent studies show that protein–protein interaction model evaluations can be skewed by train–test leakage, especially when dataset splits rely solely on sequence or structural similarity (Bushuiev *et al.* 2024). Proteins with low sequence identity may have similar local interface geometries, resulting in overly optimistic performance estimates. To address this, interface-based benchmarks like PINDER have been proposed to minimize leakage in dataset splits (see Section D for more details, available as supplementary data at *Bioinformatics* online).

One of the test sets introduced by PINDER is PINDER-S, which consists of 250 dimers (500 individual proteins), and we included PINDER-S as an additional benchmark to complement the evaluations of the MaSIF-site and P-250 sets. This benchmark is valuable for interface prediction as it reduces the risk of strong performance being due to structural redundancy between training and test interfaces. It rigorously assesses whether a model has learned transferable principles of molecular recognition, such as interface geometry and residue organization, rather than just memorizing recurring patterns from similar complexes (Bushuiev *et al.* 2024, Kovtun *et al.* 2024).

The results on the PINDER-S set are presented in Table 1. BiMba achieves the highest overall performance across several key metrics, including ACC (0.87), F1-score (0.42), Pre (0.35), area-weighted AUC (0.81), and PRC (0.35). This demonstrates a strong ability to identify true interface residues while minimizing false positives. Compared to other methods, BiMba consistently offers a better tradeoff between Pre and Rec, resulting in the best F1-score among all models. It is worth noting that while dMaSIF achieves the highest Rec (0.56) and a slightly higher AUC (0.70), this performance comes at the cost of lower precision and overall balance, leading to a reduced F1-score. BiMba maintains robust performance across various metrics, which reflects its ability to more accurately identify the true physical interface regions. These results highlight that BiMba generalizes effectively to the leakage-reduced PINDER-S set, capturing biologically meaningful interaction patterns beyond those present in the training data.

### 3.4 Performance of BiMba on proteins of different types

A deeper understanding of a model’s strengths emerges when its performance is evaluated under diverse biochemical conditions. To investigate this, we compared BiMba with other methods across biologically meaningful subsets derived from the MaSIF-test set. First, we grouped the proteins according to the hydrophobicity of their binding interfaces, distinguishing those with small versus large hydrophobic surface areas, a key determinant of interface energetics. Second, we grouped proteins by interaction type, distinguishing obligate complexes, that is, proteins that form stable, essentially permanent associations with a specific partner, from transient complexes, which interact only temporarily and often with multiple partners, as in many signaling processes. Across both categorizations, BiMba demonstrated better performance across all metrics except Pre and one instance of AUC (Table 2), demonstrating robust performance across fundamentally different interaction types. Two example proteins from the MaSIF-test set are discussed qualitatively (see Section 3.7) for strengths and weaknesses.

**Table 2:** Performance comparison of different methods across hydrophobicity region sizes and interaction types.

	Large hydrophobicity				Small hydrophobicity				Obligate interactions				Transient interactions			
Method	Rec	Pre	F1	AUC (area)	Rec	Pre	F1	AUC (area)	Rec	Pre	F1	AUC (area)	Rec	Pre	F1	AUC (area)
dMaSIF	0.30	0.41	035	0.75	0.29	**0.37**	0.25	0.74	0.35	**0.44**	0.37	0.76	0.22	**0.32**	0.26	**0.77**
MaSIF-site	0.27	0.29	0.23	0.69	0.17	0.27	0.22	0.67	0.39	0.33	0.34	0.73	0.17	0.28	0.22	0.70
HCGNet	0.39	0.38	0.33	0.74	0.39	0.25	0.28	0.73	0.25	0.41	0.35	0.69	0.23	0.21	0.19	0.71
GraphRBF	0.25	**0.51**	0.26	0.67	0.23	0.35	0.19	0.69	0.23	0.41	0.25	0.76	0.40	0.24	0.25	0.76
BiMba	**0.68**	0.37	**0.45**	**0.78**	**0.53**	0.26	**0.35**	**0.75**	**0.63**	0.43	**0.45**	**0.79**	**0.57**	0.26	**0.29**	0.73

Bold values indicate the highest values under corresponding metrics.

Because only about 10% of residues in a typical protein participate in the binding interface, binding site prediction is inherently highly imbalanced; therefore, Pre and Rec are especially important for evaluating how well a model identifies true interface residues while avoiding excessive false positives (Dai and Bailey-Kellogg 2021). However, single-threshold Pre or Rec can often be misleading because small changes in the threshold can lead to a situation where a few true positives are traded for many false positives, which can greatly affect precision. In such cases, Pre–Rec curves offer a better summary of ranking quality by explicitly focusing on the positive class and its enrichment (Saito and Rehmsmeier 2015).

**Figure 2:**
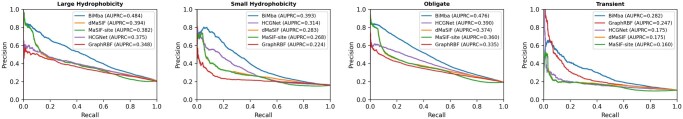
Precision–Recall curves comparing different methods across hydrophobicity region sizes and interactions.

We also report Pre–Rec curves for all compared methods across the four protein subsets. Figure 2 shows that BiMba demonstrates the strongest Pre–Rec performance across all four subsets, as indicated by its highest AUPRC. This suggests that BiMba effectively identifies true interface residues across a wide range of operating thresholds. Detailed information about each subset, including the number of proteins, residues, interface residues, and interface ratio, is provided in Section H, available as supplementary data at *Bioinformatics* online.

Large language models (LLMs) have reshaped many areas of machine learning and are increasingly influencing protein research by providing powerful sequence-based representations that capture evolutionary and structural context (Pokharel *et al.* 2025). As LLM-driven approaches gain traction in protein binding-site prediction, it becomes important to understand how alternative paradigms—such as vision-based surface modeling—compare. For this reason, we evaluated BiMba against DeepProSite (Fang *et al.* 2023), a recent LLM-based predictor, to assess whether structural properties and surface information alone can achieve comparable predictive performance. As shown in Section F, available as supplementary data at *Bioinformatics* online, BiMba achieves performance comparable to DeepProSite on several metrics and exceeds it on others, demonstrating that a surface-centric vision model can rival the predictive strength of LLM-based approaches despite relying on fundamentally different representations (see Section F for details, available as supplementary data at *Bioinformatics* online).

### 3.5 Explainability

Explainability methods in DL allow us to interpret the decisions made by the model, providing biologically relevant explanations for the predictions. In the context of protein interaction modeling, explainability is particularly important because it connects computational predictions to biological reasoning, ensuring that the model identifies functionally relevant areas rather than spurious correlations. Unlike transformers, ViM does not produce explicit attention maps, but Ali *et al.* (2025) introduced an approach to extract hidden attention values from the middle layers of Mamba. These hidden attention profiles highlight the importance of the surface features that strongly influence the final prediction.

Using the approach of Ali *et al.* (2025), we obtain point-level attention maps from the ViM encoder. We applied this method to obtain point-level attention maps. We then applied two additional techniques that elucidate the relationship between surface features and interaction propensities.

**Figure 3:**
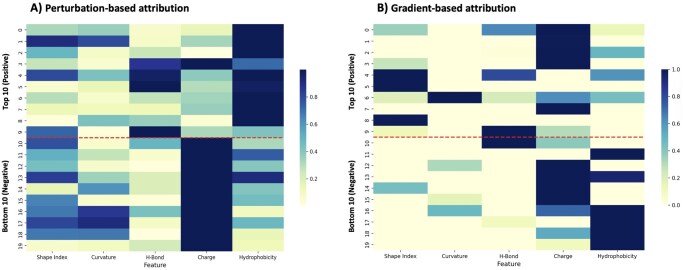
Feature-level explainability for BiMba using perturbation-based and gradient-based attribution. The top 10 highest probability (positive) points and the bottom 10 lowest probability (negative) points of protein 2V3B-A are displayed. (A) Perturbation-based attribution heatmap. Larger drops indicate greater feature reliance. (B) Gradient × Input attribution heatmap illustrating per-channel importance scores obtained by multiplying input values with the gradient of the output.

First, we applied a perturbation-based attribution scheme (Zeiler and Fergus 2014) (see Fig. 3A). We ranked all patches on the protein surface and visualized the top 10 positively predicted patches and the bottom 10 negatives for a representative protein 2V3B-A, one of the best predictions by BiMba. For each surface patch, we masked one of the five input features (channels) at a time by setting it to zero and measured the resulting drop in binding site probability. The larger the drop, the more the model relies on that feature for its decision. In this example, charge emerges as the most critical feature for correctly rejecting noninterface points (negatives), while hydrophobicity has the strongest impact for confidently predicted interface points (positives), indicating that BiMba leverages electrostatic cues to avoid false positives and hydrophobic clustering to support true interface predictions.

Second, we used a gradient-based attribution method (Ancona *et al.* 2018) to quantify feature contributions (see Fig. 3B). For each patch and channel, we computed the gradient of the output score with respect to the input and multiplied it by the input value (Grad×Input), yielding a per-channel importance score. In the same protein example, charge again appears as a key determinant for both positive and negative patches, while geometric descriptors (shape index and curvature) and hydrogen-bond feature (H-Bond) contribute more strongly to high-confidence positives. Intuitively, this suggests that, given similar biochemical context, BiMba is sensitive to how much the local surface shape and hydrogen-bonding environment must change to flip a point from interface-like to noninterface-like, or vice versa. This level of feature interpretability is particularly valuable in protein design, as it shows which geometric or physicochemical modifications to the surface would most effectively strengthen or weaken interaction propensities.

### 3.6 Comparing with AlphaFold3

AlphaFold3 (Abramson *et al.* 2024) represents a major advance in predicting biomolecular structures, extending its modeling capabilities from individual proteins to a diverse range of biomolecular complexes. It can derive plausible 3D arrangements directly from sequences, and when additional molecular context is provided, this capability enhances its effectiveness. As a result, AlphaFold3 has become a widely recognized benchmark for predicting the structure of biomolecular complexes involving more than one molecule. However, it is important to note that AlphaFold3 is primarily designed for structure prediction; identifying binding sites at the residue level is typically performed indirectly by postprocessing the predicted complex geometries to infer interchain contacts. To perform a comparison (as fairly as possible) between BiMba and AlphaFold3, we created a set of 47 protein–protein complexes (94 proteins) drawn from the P-250-test set, where each complex corresponds to a unique protein pair without reuse of proteins across different complexes.

For each predicted model, we identified residue labels by detecting interchain contacts based on a 5-Å distance cutoff. For each individual protein contributed to the protein complex, residues that engaged in at least one interchain contact were classified as interface residues, while all other residues were labeled as noninterface.

We evaluated the predictions from AlphaFold3 by using only the top-ranked model provided by the AlphaFold3. Additionally, we performed the comparison by averaging across the five highest-ranked models for each protein, but it did not differ substantially from that of using the best model across all metrics. Results are provided in Table 3.

**Table 3:** Performance comparison of BiMba and AlphaFold3.

Method	ACC	Rec	Pre	F1	AUC	PRC
AlphaFold3	0.92	**0.66**	**0.65**	**0.64**	0.81	**0.55**
BiMba	**0.93**	0.58	0.54	0.57	**0.84**	0.53

Bold values indicate the highest values under corresponding metrics.

Table 3 indicates that AlphaFold3 demonstrates higher recall and precision, resulting in an improved F1-score. This suggests that when AlphaFold3 successfully predicts a protein–protein complex, it is effective in accurately inferring the interfaces and, consequently, the binding sites of each individual protein within the complex. BiMba demonstrates improved accuracy and AUC, indicating its superior ability to distinguish between interface and noninterface residues throughout the dataset.

However, AlphaFold3 requires the presence of both interacting partners and infers binding site residues for each individual protein indirectly from predicted complex geometries. In contrast, BiMba is a partner-agnostic binding site predictor that can be applied either to full protein complexes or to individual proteins in isolation. As a result, even with powerful tools such as AlphaFold3, complementary methods like BiMba remain necessary, as they provide broader applicability in scenarios where interaction partners are unknown, incomplete, or variable—such as proteins whose interaction partners vary depending on cellular state, modification, or biological context.

Moreover, despite its strong average performance, AlphaFold3 fails to identify correct binding site residues for 12 out of 94 proteins, yielding zero recall and F1-score for those samples. In contrast, BiMba is still able to identify interface residues for these proteins, at least partially, as shown in Table 4. Further evaluation on a much larger test set is included in Section E, available as supplementary data at *Bioinformatics* online.

**Table 4:** Performance of BiMba on proteins for which AlphaFold3 was unable to identify interface residues.

Protein	ACC	Rec	Pre	F1	AUC	PRC
3bx1_A	0.93	0.10	0.99	0.14	0.60	0.17
2vlu_A	0.77	0.83	0.30	0.44	0.83	0.39
1yla_A	0.87	0.45	0.31	0.37	0.87	0.28
1u9b_A	0.86	0.29	0.24	0.26	0.57	0.14
1iko_A	0.52	0.80	0.16	0.26	0.63	0.15
3etp_A	0.40	1.00	0.12	0.20	0.67	0.15
1kcq_A	0.52	0.58	0.36	0.43	0.62	0.17
2ghv_A	0.29	1.00	0.16	0.27	0.59	0.15
1coo_A	0.88	0.95	0.50	0.17	0.55	0.19
4i8a_AB	0.75	0.13	0.19	0.16	0.58	0.14
1few_A	0.17	1.00	0.11	0.20	0.33	0.07
4dsr_AB	0.95	0.23	0.11	0.20	0.56	0.25

### 3.7 Two sample proteins—a qualitative discussion

**Figure 4:**
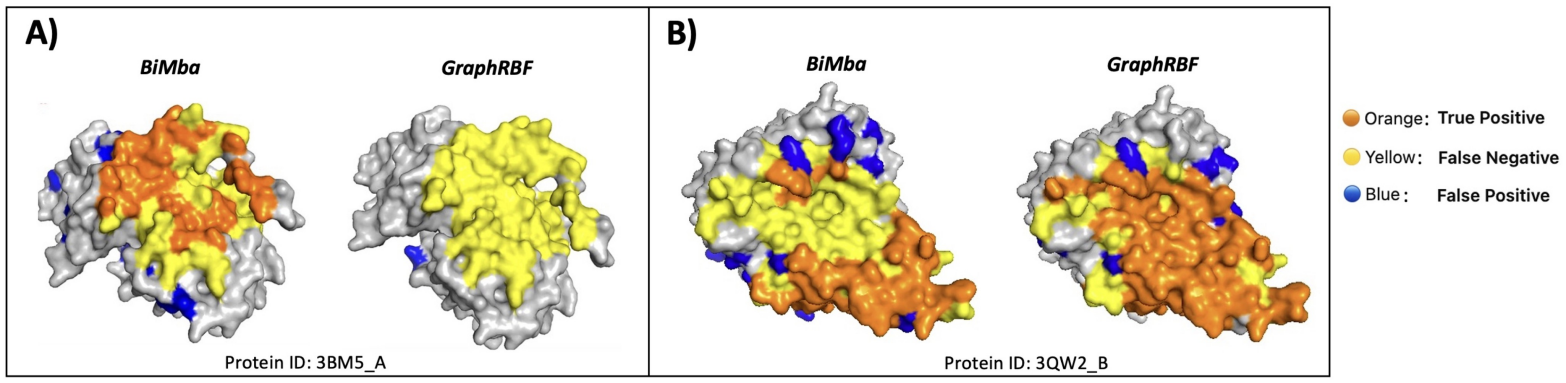
Qualitative comparison of interface predictions by BiMba and GraphRBF on two representative proteins from the MaSIF-test set, where orange indicates true positives, yellow indicates false negatives, and blue indicates false positives. (A) Protein 3BM5-A, where BiMba achieves one of its best predictions, accurately recovering most of the true interface (orange) with few missed residues (yellow), whereas GraphRBF fails to detect the majority of interface residues. (B) Protein 3QW2-B, representing one of GraphRBF’s best-performing cases, where it correctly identifies most interface residues; BiMba still captures a substantial portion of the interface, though with more false negatives.

Figure 4 illustrates qualitative predictions for two representative proteins from the MaSIF-test set. The left example (3BM5-A) (Fig. 4A) corresponds to the case where BiMba achieves its best prediction, correctly recovering most of the experimental interface as a compact orange region with relatively few missed residues (yellow). In contrast, GraphRBF largely failed on this target, leaving the majority of interface residues as false negatives. For the right example (3QW2-B)—the best prediction produced by GraphRBF (Fig. 4B)—the method covered almost the entire binding site with true positives, while BiMba still identified a substantial portion of the interface, though with more false negatives. These examples highlight that BiMba tends not to completely miss interfaces—even on targets where it is not the top-performing method—whereas GraphRBF can perform extremely well on certain proteins but may fail dramatically on others.

### 3.8 Ablation study

To understand the contribution of each component in BiMba, we conducted a systematic ablation study on the MaSIF-test set (see Table 5). The results show that: (i) converting surface patches into image-like representations provides a richer and more expressive encoding than point-based convolutional features; (ii) Mamba-based architecture produced an additional performance gain (3% over ViT, 4% over MaSIF-site), demonstrating the advantage of bidirectional state-space modeling for capturing long-range surface patterns and spatial continuity on protein interfaces; (iii) a CNN-based model using the same 2D representation achieves lower performance than both ViT- and Mamba-based models, indicating that architecture plays a key role beyond model scale; (iv) smart use of local and global residue-level features improves the performance, and finally, (v) Combining both local and global residue-level features with the 2D surface grids resulted in the highest AUC (0.88), corresponding to a total improvement of 10% over the MaSIF-site baseline and 6% over the Mamba Base (BiMba [Base]) (see Section A for more details, available as supplementary data at *Bioinformatics* online).

**Table 5:** Ablation study showing the performance of different methods on the MaSIF test set.

Model	Improvement	Parameters	AUC (area)
MaSIF-site	None	66 080	0.78
CNN-based	2D surface maps	66 954	0.74
ViT-based	2D surface maps	2 865 418	0.79
BiMba (Base)	Mamba-based architecture	1 038 772	0.82
BiMba (Base) local features	RD, PI, CN, SS, AP	1 042 084	0.85
BiMba (Base) + global features	RSA, residue polarity and residue name	1 042 052	0.86
BiMba	2D surface maps + all residue-level features	1 046 196	**0.88**

RD, residue depth; PI, protrusion index; CN, coordination number; SS, secondary structure; AP, amide plane; RSA, relative solvent accessibility.Bold values indicate the highest values under AUC (area).

## 4 Discussion

BiMba consistently achieved strong and well-balanced performance in most classification metrics across different test sets. BiMba often outperformed state-of-the-art graph-based methods such as GraphRBF and geometric surface methods such as MaSIF-site and dMaSIF, showing improvements in interface localization metrics. BiMba also generalized effectively across proteins of different sizes, hydrophobicity profiles, and interaction types (obligate and transient), highlighting its robustness to biochemical and structural variability.

Beyond predictive performance, BiMba provides interpretable outputs. Using hidden attention extraction across Mamba layers, we demonstrated how individual surface features contribute to the model decisions. These explanations not only validate that BiMba relies on biologically meaningful cues but also provide actionable insights for protein design, revealing which geometric or physicochemical modifications to the surface would most strongly affect binding propensity.

Overall, our results show that modern vision-based architectures, and particularly Mamba-style bidirectional SSMs, offer a powerful and efficient alternative to transformers and graph neural networks for protein interface prediction. The findings of this study further reinforce the findings of (Shirali *et al.* 2025b), showing that Mamba-based models can surpass transformer architectures in capturing long-range spatial cues and protein surface-dependent interaction patterns. The results of the ablation study in Section 3.8 support these claims with clear improvements, quantifying the contributions of image representations, Mamba architecture, and a combination of novel local and global residue-level features.

From a biological perspective, our findings reinforce the central importance of surface information in governing protein interactions and demonstrate that integrating visual surface patterns with residue-level descriptors can substantially improve binding site identification. We anticipate that BiMba will contribute not only to interaction prediction tasks but also to downstream applications in protein engineering and rational design, where both accuracy and interpretability are essential.

We also highlight the strengths and limitations of recent breakthroughs in machine learning for structural biology by comparing BiMba with AlphaFold3. While AlphaFold3 demonstrates high performance in certain classification metrics, our analysis shows that relying solely on high-performance foundation models does not eliminate the need for specialized, task-focused methods. Specifically, AlphaFold3 is not designed to directly predict binding sites for an individual protein in isolation, a common scenario in protein engineering, partner design, and early stage drug discovery, where the interacting partner may be unknown or still under development. In contrast, BiMba can predict binding sites without requiring a known binding partner or a predefined complex, even in examples where AlphaFold3 struggles. The development of hybrid frameworks that integrate AlphaFold3’s powerful structural insights with BiMba’s detailed surface-level reasoning could be a promising direction for future work.

A few thoughts on the limitations of our method. Our current approach assumes proteins to be rigid structures, overlooking the flexibility and conformational variability that naturally occur during molecular interactions. However, in nature, a protein can adapt its surface shape when binding to different partners, and such induced-fit or conformational selection mechanisms are biologically significant (Teague 2003, Csermely *et al.* 2010). Capturing this flexibility and incorporating it into our model would enable the model to more accurately reflect the true dynamic nature of protein during interaction, ensuring that the predicted binding site more closely represents what occurs in biological systems. The primary obstacle lies in the lack of large-scale, high-quality datasets containing multiple conformations of the same protein bound to various partners. Future efforts could integrate data from molecular dynamics simulations or experimental ensemble structures to introduce conformational diversity into the learning process.

Another limitation arises when a protein possesses multiple distinct binding sites. Our current framework is trained primarily to identify a single dominant interface that may overlook secondary or allosteric binding regions. Biologically, these additional sites can play critical roles in regulation, signaling, or cooperative binding, where different partners interact with the same protein at separate locations or under different conditions (Keskin *et al.* 2008, Vajda *et al.* 2017). This enhancement would make the framework more biologically realistic and useful for studying proteins involved in multipartner or allosteric interactions.

In addition, we intend to examine our model’s performance on peptides, which represent a more challenging case for binding-site prediction. Unlike large, globular proteins, peptides are short, flexible, and often lack well-defined tertiary structures, making it more difficult to identify stable binding interfaces (Petsalaki and Russell 2008, Vanhee *et al.* 2011). Testing BiMba and competing tools on this dataset will provide a more rigorous assessment of robustness and generalization, revealing how well each approach captures biologically meaningful interaction patterns across proteins of varying size and structural complexity.

## 5 Conclusions

BiMBa uses a Vision Mamba–based framework for protein binding site prediction. By adapting state space models to protein surface data, BiMba learns spatial and physicochemical features, enabling accurate and robust identification of interaction-prone regions. Our design integrates both surface-level representations and complementary residue-level descriptors. Surface-level representations capture geometric and physicochemical properties such as shape, electrostatics, and hydrophobicity. Residue-level descriptors capture information about each residue, such as polarity, type, solvent accessibility, and secondary structure. Previous efforts focused on one feature or another; we combined the broadest set of features to achieve improved performance. Careful selection of biochemically important points, and the explainability capabilities add significance to the results.

## Supplementary Material

btag243_Supplementary_Data

## Data Availability

*The data underlying this article are available in the article and in its online supplementary material.*
