# Supplementary material for: BiMba: using Vision Mamba to predict protein sites that bind other proteins
Source: Bioinformatics. 2026 Jul 7;42(Suppl 1):btag243. doi: 10.1093/bioinformatics/btag243 (PMC13340168; doi:10.1093/bioinformatics/btag243)
Supplement: btag243_Supplementary_Data [file btag243_supplementary_data.pdf]

BiMba: Using Vision Mamba to Predict Protein Sites that Bind Other Proteins

A. Shirali et al.

Supplementary materials

A Ablation study

To understand the contribution of each component in **BiMba**, we conducted a systematic ablation study on the MaSIF-test set (Table S1). Starting from the MaSIF-site baseline, which relies on a convolutional Siamese architecture operating directly on point-level geometric descriptors, we first evaluated a CNN-based model trained on our 2D surface grids, which resulted in lower performance, indicating that a standard convolutional architecture is not sufficient to fully exploit the proposed representation. We then replaced this pipeline with a ViT-based model trained on our 2D surface grids.

Next, we introduced the Vision Mamba encoder to form the **BiMba** (Base) model, keeping the inputs identical to the ViT baseline. The Mamba-based architecture produced a clear performance gain (3% over ViT, 4% over MaSIF-site), demonstrating the advantage of modeling long-range dependencies.

We then augmented the base model with local residue-level features (residue depth, protrusion index, coordinate number, secondary structure, amide plane), which improved performance further (3% over **BiMba** (Base)) by providing fine-grained structural context around each surface patch. Then, we only add global residue-level features (relative solvent accessibility, polarity, residue type), which produced an additional improvement (4% over **BiMba** (Base)), showing that global biochemical context complements local structural cues.

Finally, combining both local and global residue-level features with the 2D surface grids resulted in the full **BiMba** model, which achieved the highest AUC (0.88), corresponding to a total improvement of 10% over the MaSIF-site baseline and 6% over the Mamba Base (**BiMba** (Base)). This progression highlights that each component—2D surface maps, the Mamba encoder, local descriptors, and global biochemical descriptors—contributes incrementally to performance, with the complete **BiMba** framework providing the most accurate and comprehensive binding-site predictions. Moreover, the CNN-based model demonstrates lower performance, even though it has a similar number of parameters to MaSIF-site. In contrast, both the ViT and Mamba-based models show improved performance, with Mamba surpassing ViT despite having fewer parameters. This suggests that the enhancements are not solely dependent on the size of the model but rather on the architectural design. Specifically, Mamba effectively captures long-range dependencies through state-space modeling, which is more efficient compared to the attention-based approach used in ViT.

B Surface Point Selection Strategy

B.1 Training phase:

Proteins contain thousands of surface vertices (points) even at moderate sizes. Consequently, during training, we aimed to achieve computationally efficient yet biologically meaningful sampling of surface points while ensuring that every residue contributes to the learning process. We subsampled surface points on a per-residue basis. For residues containing both interface (iface = 1) and non-interface (iface = 0) vertices, points from each group were retained proportionally to capture the surface contrast entirely. For residues with only non-interface vertices, a subset (10%) was randomly selected. This sampling approach ensures that: 1- Interface residues are fully represented, capturing their geometric and physicochemical variability. 2- Non-interface residues are included in a proportional manner to summarize background topography.

Table S1: Ablation study showing the performance of different methods on MaSIF-test set.

Model	Improvement	Parameters	AUC (area)
MaSIF-site	None	66,080	0.78
CNN-based	2D surface maps	66,954	0.74
ViT-based	2D surface maps	2,865,418	0.79
**BiMba** (Base)	Mamba-based architecture	1,038,772	0.82
**BiMba** (Base)+ local features	Residue depth, Protrusion index, Coordinate number, Secondary structure, Amide plane	1,042,084	0.85
**BiMba** (Base)+ global features	Relative solvent accessibility, Residue polarity, Residue name	1,042,052	0.86
**BiMba**	2D surface maps & all residue-level features	1,046,196	**0.88**

Bold values indicate the highest values under corresponding metrics.

B.2 Inference phase:

For inference, we employed a deterministic, atom-prioritized selection strategy to identify a representative subset of surface points for each residue. The rationale was to select vertices (points) of the atoms that are most indicative of a residue's potential contribution to a binding site. The following hierarchical selection strategy was used:

1. **Side-chain heteroatoms (primary selection):** If the residue contained polar or charged atoms capable of hydrogen bonding or electrostatic interactions, surface points associated with these atoms were selected first. These atoms dominate the specificity of protein–protein and protein–ligand interfaces by forming directional hydrogen bonds and salt bridges.

2. **Aromatic side-chain carbons (secondary selection):** For residues lacking polar side chains, aromatic carbons were selected next. These atoms participate in $\pi - \pi$ stacking and π–cation interactions, which are critical for interface stability and hydrophobic packing.

3. **Backbone carbonyl and amide atoms (tertiary selection):** If no side-chain atoms were available, backbone atoms O (carbonyl oxygen), OXT (C-terminal oxygen), and N (amide nitrogen) were chosen. These atoms frequently mediate hydrogen bonds in β-sheet or helix–helix interfaces and act as secondary contributors to interfacial recognition when side chains are buried or absent.

4. **Aliphatic heavy atoms (final selection):** If the residue lacked both polar and aromatic atoms, the vertices (points) of aliphatic carbons (CB, CG, CD1, or CD2) are selected. These atoms are typically hydrophobic and help form the nonpolar core of protein–protein interfaces that stabilize the overall complex.

The biochemical rationale behind this strategy is that while hydrophobic residues contribute substantially to the binding free energy by forming the buried nonpolar core of protein–protein interfaces, they primarily stabilize the complex rather than determine its specificity. In contrast, polar heteroatoms define the directional hydrogen-bonding and electrostatic patterns that govern molecular recognition and partner selectivity. This polar $\longrightarrow$ aromatic $\longrightarrow$ backbone $\longrightarrow$ hydrophobic order mirrors the established hierarchy of interaction propensities in natural interfaces Jones and Thornton (1997); Chakrabarti and Janin (2002); Keskin et al. (2008); Levy (2010); Chothia and Janin (1975).

To evaluate the effectiveness of our point selection strategy during inference, we conducted an ablation study where surface points were chosen randomly instead. The objective of this experiment was to quantify the potential mismatch between the training sampling strategy and the inference-time selection method. The results presented in Table S2 indicate a significant decline in performance when random selection is employed, with the AUC dropping from 0.88 to 0.66 and the F1-score decreasing from 0.44 to 0.23. This indicates that selecting representative surface points during inference is crucial for preserving biologically meaningful interaction patterns. The proposed selection strategy prioritizes atoms that are more likely to be involved in molecular recognition, thus maintaining important geometric and physicochemical signals. In contrast, random sampling introduces noise and dilutes these signals. These findings demonstrate that a deterministic, biochemically guided selection process is essential for achieving robust and accurate predictions of binding sites.

Table S2: Impact of point selection strategy during inference on model performance.

Point Selection	ACC	Rec	Pre	F1	AUC	AUC (area)	PRC
Random	0.65	0.26	0.20	0.23	0.66	0.69	0.22
Proposed Strategy	**0.83**	**0.56**	**0.36**	**0.44**	**0.88**	**0.88**	**0.41**

Bold values indicate the highest values under corresponding metrics.

C Residue-level features

To complement the 2D surface grids used in **BiMba**, we extracted a set of residue-level descriptors that capture both local geometric context and global biochemical properties around each surface point. These features provide structural cues that are not fully encoded in surface patches alone and were shown to enhance model performance substantially. The brief definitions of each residue-level feature used in this work are as follows.

- **Secondary structure:** For each residue, we assign an eight-state secondary-structure label (helix, strand, turn, coil, etc.) obtained from DSSP Touw et al. (2015). Secondary structure encodes the local 3D conformation around the $C\alpha$ atom and captures recurrent backbone motifs that are often enriched or depleted at protein–protein interfaces.
- **Residue depth:** Residue depth measures how far a residue lies beneath the solvent-accessible surface, defined as the average distance of its atoms from the molecular surface. Shallow residues have small depth and lie near the exterior, whereas large depth values correspond to buried residues; Residue depth complements RSA by capturing how deeply a residue is embedded within the protein interior.
- **Protrusion index:** The protrusion index quantifies how much a residue sticks out from the surrounding protein environment. We approximate the protrusion index using a CX-value–style measure inspired by Kahraman et al. Kahraman et al. (2007), by counting local $C\alpha$ contacts within a fixed-radius neighborhood and converting this contact pattern into a normalized exposure score. High protrusion index values indicate locally exposed or bulging residues that are more likely to participate in binding.
- **Coordinate number:** Coordinate number measures the local contact density around a residue. It is defined as the number (or weighted count) of neighboring residues whose $C\alpha$ atoms lie within a distance at which their interaction is considered significant. Residues with high coordinate numbers reside in densely packed regions, while low-coordinate number residues are more isolated or located on protruding surface regions Morehead et al. (2023).
- **Amide normal vector:** For each residue, the amide normal vector is the unit vector perpendicular to the peptide plane formed by backbone atoms ($C\alpha$, $C\beta$, and N). This vector encodes the local orientation of the backbone and side chain and can be used to reason about angular relationships between neighboring residues, for example when characterizing how backbone orientation changes across an interface.
- **Relative Solvent Accessibility (RSA):** RSA is the fraction of a residue's solvent-accessible surface area that is exposed to water, normalized by a residue-specific maximum accessibility. High RSA indicates surface-exposed residues, whereas low RSA indicates buried residues; together with hydrophobicity, this helps distinguish residues likely to participate in binding from core or fully solvent-exposed sites.
- **Polarity:** This feature characterizes whether a residue's side chain is polar, nonpolar, or charged, reflecting its tendency to engage in hydrogen bonding, electrostatic interactions, or hydrophobic packing. This descriptor groups amino acids based on their physicochemical behavior (e.g., polar uncharged, positively charged, negatively charged, hydrophobic), providing the model with high-level biochemical context that complements geometric features. Including polarity helps **BiMba** recognize chemically distinct regions on the protein surface and improves its ability to identify residues more likely to participate in binding interactions.
- **Residue type (one-letter code):** Each amino acid is represented by its standard one-letter identifier (e.g., A, R, N, D). This categorical descriptor encodes the intrinsic biochemical identity of the residue, capturing properties such as charge, hydrophobicity, polarity, and aromaticity. Including residue type provides the model with fundamental chemical context that complements geometric features, allowing **BiMba** to distinguish residue classes that differ in their likelihood of participating in protein binding sites Xue et al. (2015); Talavera et al. (2011).

D Evaluation on PINDER-S benchmark

Recent studies indicate that the evaluation of protein–protein interaction (PPI) models can be significantly influenced by train–test leakage when dataset splits are created solely based on metadata or sequence similarity. This issue is especially concerning from a biological perspective, as proteins with different overall structures or low sequence identity can still share highly similar local interface geometries. As a result, test examples may end up being too similar to those in the training set. Bushuiev et al. Bushuiev et al. (2024) addressed this issue by introducing iDist, a scalable approximation to interface-alignment methods that is designed to directly compare protein–protein interfaces in 3D rather than relying on sequence or metadata proxies. They demonstrated that metadata-based and sequence-based splits can lead to significant leakage, while interface-based comparisons provide a more accurate estimate of generalization to unseen binding modes.

In a separate study, Bushuiev et al. Bushuiev et al. (2023) introduced PPIRef, a comprehensive collection of non-redundant protein-protein interfaces sourced from the Protein Data Bank. They utilized iDist to efficiently eliminate structurally similar interfaces. The core idea they presented is biologically significant: when evaluating generalization in PPI learning, the focus should not solely be on the overall protein structure but also on the interaction interface itself. This is because molecular recognition is determined by the shape and physicochemical properties of the contacting regions.

Building on previous work, PINDER has been proposed as a comprehensive structural benchmark and evaluation resource for PPIs. PINDER begins with biological assemblies from the RCSB NextGen database and breaks them down into binary PPIs, annotating them with interface-related structural information. Notably, PINDER was specifically designed to minimize leakage between training, validation, and test sets by integrating structural similarity (using Foldseek) and sequence similarity (using MMseqs2), while focusing on the construction of splits based on interface residues. In addition to bound complexes, PINDER also incorporates apo structures and predicted models, providing a more realistic resource for assessing generalization in the context of biologically relevant conformational variations. The authors further demonstrated that models evaluated on the de-leaked PINDER splits can perform significantly worse than those tested on conventional, more leaky benchmarks. This highlights the crucial impact that benchmark design can have on perceived progress in the field Kovtun et al. (2024)

E Comparing with AlphaFold3 on Dockground benchmark

To further assess the performance of **BiMba** compared to AlphaFold3, we conducted additional experiments using a larger and more diverse benchmark. This involved datasets obtained from the Dockground resource, which offers extensive, curated collections of protein–protein complexes derived from experimentally validated structures. Dockground is widely recognized as a valuable tool for evaluating docking and interface prediction methods. Specifically, we utilized unbound docking benchmark set 4, which includes 396 non-redundant complexes comprising 792 individual proteins Kundrotas et al. (2018).

Furthermore, the P-250 set used in our experiments is derived from protein–protein interaction datasets curated from Docking Benchmark 5.5 (DBD 5.5) Vreven et al. (2015) and Dockground Kundrotas et al. (2018). As such, the Dockground dataset serves as an excellent extension of P-250, offering a larger and more comprehensive collection of complexes while remaining consistent with the original data source. This motivates our selection of Dockground for further evaluation of our model against AlphaFold3, allowing for a more extensive assessment.

Table S3: Performance comparison of **BiMba** and AlphaFold3 on Dockground set.

Method	ACC	Rec	Pre	F1	AUC	PRC
AlphaFold3	0.91	**0.62**	**0.63**	**0.62**	0.79	**0.53**
**BiMba**	**0.93**	0.57	0.47	0.58	**0.82**	0.49

Bold values indicate the highest values under corresponding metrics.

The performance comparison between **BiMba** and AlphaFold3 on the Dockground benchmark is presented in Table S3. **BiMba** achieves a higher accuracy of 0.93 compared to 0.91 for AlphaFold3, as well as a better area under the curve (AUC) of 0.81 versus 0.79. On the other hand, AlphaFold3 shows better recall (0.62 vs. 0.57), precision (0.63 vs. 0.47), F1-score (0.62 vs. 0.58), and precision-recall curve (PRC) (0.53 vs. 0.49). These metrics reflect AlphaFold3's strength in recovering known interfaces when both interacting partners are provided.

Although **BiMba** is not specifically designed as a model for predicting interactions, it demonstrates competitive performance and even outperforms AlphaFold3 in global discrimination metrics such as AUC. These results highlight

BiMba's ability to effectively capture interaction-relevant patterns from protein surfaces. In contrast, AlphaFold3 benefits from explicit pairwise structural modeling.

Table S4 includes a list of proteins for which AlphaFold3 was unable to predict interface residues. It shows that AlphaFold3 could not identify interface residues in 58 proteins, while **BiMba** was able to partially recover some interaction-relevant regions, although in some instances, only a small portion of the interface was identified. On the other hand, there are 23 proteins where AlphaFold3 accurately predicted interface residues, but **BiMba** did not, demonstrating the advantage of AlphaFold3 when explicit partner information is available. Additionally, there are 16 proteins for which both methods failed to identify interface residues, highlighting particularly challenging cases that neither approach was able to capture effectively.

These observations indicate that **BiMba** and AlphaFold3 capture different but complementary aspects of protein–protein interactions. Therefore, the two approaches can effectively complement each other in practice. However, the existence of cases where both methods fail highlights the need for further research and improvements in the methodologies used for predicting protein binding sites.

Table S4: Performance of **BiMba** on proteins for which AlphaFold3 was unable to identify interface residues on Dockground set.

**Protein**	**ACC**	**Rec**	**Pre**	**F1**	**AUC**	**PRC**
5e8f_A	0.84	0.26	0.36	0.30	0.63	0.22
2jrh_A	0.47	0.88	0.25	0.39	0.60	0.25
1om0_A	0.72	0.60	0.15	0.24	0.69	0.14
2gy5_A	0.68	0.53	0.06	0.11	0.56	0.05
2btd_A	0.38	0.85	0.22	0.35	0.52	0.21
4orb_AB	0.95	0.05	0.17	0.07	0.50	0.05
1h9v_A	0.47	0.91	0.18	0.31	0.70	0.21
1ga3_A	0.81	0.43	0.50	0.46	0.74	0.42
3g6a_AB	0.84	0.24	0.20	0.22	0.50	0.14
1iko_A	0.76	0.13	0.25	0.17	0.48	0.19
3etp_A	0.79	0.50	0.45	0.48	0.72	0.35
4k0a_AB	0.72	0.41	0.19	0.26	0.67	0.18
1u9b_A	0.63	0.56	0.21	0.31	0.59	0.16
1yla_A	0.57	0.71	0.26	0.38	0.70	0.29
1f7t_ABC	0.89	0.28	0.62	0.39	0.73	0.31
3eps_A	0.81	0.31	0.14	0.19	0.63	0.10
3poa_A	0.58	0.68	0.15	0.25	0.66	0.16
3otv_A	0.73	0.26	0.10	0.14	0.51	0.07
1vh4_AB	0.98	0.24	0.57	0.33	0.85	0.25
2hyw_A	0.49	0.84	0.14	0.23	0.73	0.20
3l88_ABC	0.81	0.42	0.08	0.14	0.63	0.06
2io4_AB	0.95	0.03	0.20	0.05	0.54	0.13
1few_A	0.79	0.50	0.38	0.43	0.81	0.38
2qts_ABC	0.97	0.15	0.29	0.20	0.72	0.20
1cl0_AB	0.88	0.49	0.42	0.45	0.74	0.33
1c3d_A	0.89	0.25	0.14	0.18	0.67	0.11
1a3s_A	0.80	0.40	0.12	0.18	0.60	0.11
2mw5_A	0.82	0.14	0.03	0.05	0.42	0.03
2ok5_A	0.82	0.35	0.14	0.20	0.60	0.09
4ehe_AB	0.84	0.38	0.18	0.24	0.62	0.13
3caf_A	0.77	0.27	0.07	0.11	0.51	0.06
2uus_A	0.72	0.54	0.13	0.21	0.63	0.12
1m7b_A	0.74	0.36	0.15	0.21	0.58	0.10
1m0z_A	0.76	0.19	0.04	0.07	0.46	0.04
1alu_A	0.67	0.56	0.19	0.28	0.65	0.17
3vx3_AB	0.88	0.44	0.30	0.36	0.74	0.29
4lw2_AB	0.58	0.73	0.10	0.17	0.61	0.10

Continued on next page

Protein	ACC	Rec	Pre	F1	AUC	PRC
1a0b_A	0.83	0.13	0.18	0.15	0.54	0.14
1fqw_A	0.32	1.00	0.11	0.20	0.44	0.08
5ea8_ABC	0.87	0.09	0.09	0.09	0.47	0.08
4v0j_AB	0.93	0.05	0.13	0.08	0.53	0.07
5dvz_AB	0.94	0.03	0.10	0.04	0.33	0.05
3pvu_ABC	0.76	0.21	0.27	0.24	0.67	0.20
2ii0_A	0.84	0.12	0.06	0.08	0.51	0.05
1csu_A	0.18	1.00	0.09	0.17	0.48	0.10
1o5r_A	0.71	0.25	0.05	0.09	0.49	0.04
4a5s_AB	0.65	0.58	0.06	0.11	0.65	0.05
4j96_A	0.85	0.20	0.22	0.21	0.68	0.14
3sbt_AB	0.73	0.29	0.25	0.27	0.51	0.17
4be8_A	0.87	0.44	0.13	0.20	0.68	0.18
4i8a_AB	0.88	0.23	0.39	0.29	0.67	0.25
3bx4_AB	0.89	0.24	0.17	0.20	0.59	0.14
2ghv_A	0.79	0.44	0.09	0.14	0.55	0.07
1qma_AB	0.85	0.13	0.17	0.15	0.54	0.14
1hge_ABCDEF	0.78	0.17	0.25	0.20	0.67	0.18
4p13_ABCD	0.78	0.17	0.06	0.09	0.45	0.05
3skn_AB	0.77	0.22	0.17	0.19	0.57	0.13
1ww9_ABC	0.80	0.16	0.22	0.19	0.58	0.15

F Comparing with DeepProSite

DeepProSite Fang et al. (2023) predicts protein binding sites by combining sequence-derived and structure-based information within a unified graph-learning framework. It first uses ESMFold Lin et al. (2023) to predict 3D protein structures directly from amino acid sequences, eliminating the need for multiple sequence alignments. From these predicted structures, the model extracts DSSP-based structural descriptors such as relative solvent accessibility, secondary structure, and backbone torsion angles. These are combined with ProtTrans Elnaggar et al. (2021) pre-trained language model embeddings that capture residue-level contextual (and implicitly evolutionary) information. Each protein is then represented as a residue-level graph, where nodes correspond to residues and edges encode spatial proximity, direction, orientation (via radial basis and quaternion functions), as well as sequence-distance information. A Graph Transformer aggregates information from neighboring residues to learn spatial and sequential dependencies, and the final multilayer perceptron outputs residue-wise probabilities of being part of a binding site. In this way, DeepProSite formulates binding site prediction as a node classification task on a structure-informed residue graph. Despite its innovation, DeepProSite's reliance on predicted structures introduces performance sensitivity to the quality of ESMFold predictions, where lower structural accuracy can reduce downstream binding site prediction accuracy.

To ensure a fair and comprehensive comparison with recent LLM-enhanced binding-site prediction models, we additionally evaluated **BiMba** on the Pro-Test-315 benchmark introduced in DeepProSite Table S5. Pro-Test-315 was specifically designed to assess models that integrate sequence language models (e.g., ProtTrans, ESMFold-derived structures) together with structural descriptors. Results for MaSIF-site, and DeepProSite are from Fang et al. Fang et al. (2023). Evaluating **BiMba** on the same dataset enables a direct comparison with DeepProSite and allows us to examine whether a vision-based surface model can match or surpass methods that rely heavily on pretrained protein language models.

In this benchmark (Table S5), **BiMba** achieves the highest accuracy (0.85) and the highest F1 score (0.47), demonstrating the strongest overall balance between detecting interface residues and avoiding false positives. Its recall (0.61) is also the highest among all methods, surpassing DeepProSite (0.58) and MaSIF-site (0.59), indicating that **BiMba** recovers a greater fraction of true interface residues. **BiMba** 's AUC (0.78) is comparable to MaSIF-site and close to DeepProSite (0.81), while maintaining a PRC (0.39) similar to dMaSIF and higher than GraphRBF. Although HCGNet attains the highest precision (0.43), this comes with lower recall and F1, reflecting a more conservative prediction pattern. Taken together, these results show that **BiMba** performs competitively even on a benchmark originally designed for LLM-enhanced models, achieving the best overall classification balance and outperforming several transformer- and graph-based baselines without relying on large-scale sequence pretraining.

Table S5: Performance comparison of different methods on Pro-Test-315 Set.

**Method**	**ACC**	**Rec**	**Pre**	**F1**	**AUC**	**PRC**
MaSIF-site	0.76	0.59	0.32	0.42	0.78	0.37
dMaSIF	0.82	0.54	0.38	0.44	0.79	0.40
HCGNet	0.80	0.44	**0.43**	0.41	0.74	**0.44**
GraphRBF	0.67	0.31	0.24	0.23	0.57	0.29
DeepProSite	0.80	0.58	0.38	0.46	**0.81**	0.43
**BiMba**	**0.85**	**0.61**	0.35	**0.47**	0.78	0.39

Bold values indicate the highest values under corresponding metrics.

G Comparing with GraphRBF

The main shortcomings of GraphRBF lies in the limited diversity and depth of features. It does not integrate surface-based or dynamic features, such as solvent accessibility, curvature, polarity, or conformational flexibility, which are biologically essential for identifying realistic interaction sites. Moreover, the model focuses on static local geometry and neglects global structural context and multi-scale dependencies between residues. As a result, its representation struggles to distinguish between true binding regions and geometrically similar but nonfunctional patches–an issue that could be mitigated by combining image-like surface projections or vision-based embeddings with graph-level reasoning. The ablation study results shown in Table S1 supports these claims.

H Test sets details

Table S6: Statistics of different hydrophobicity region sizes and interaction types subsets.

**Subset**	**#Proteins**	**#Residues**	**#Interface residues**	**Interface ratio**
Large Hydrophobicity	74	20,145	4,131	0.206
Small Hydrophobicity	74	20,060	3,139	0.156
Obligate	272	74,918	14,410	0.192
Transient	53	14,179	1,480	0.104

Interface ratio is defined as the fraction of interface residues over total residues in each subset.

References

Bushuiev, A. et al. Learning to design protein-protein interactions with enhanced generalization. *arXiv:2310.18515*, 2023.

Bushuiev, A. et al. Revealing data leakage in protein interaction benchmarks. *arXiv:2404.10457*, 2024.

Chakrabarti, P. and Janin, J. Dissecting protein–protein recognition sites. *Proteins: Structure, Function, and Bioinformatics*, 47(3):334–343, 2002.

Chothia, C. and Janin, J. Principles of protein–protein recognition. *Nature*, 256(5520):705–708, 1975.

Elnaggar, A. et al. ProtTrans: toward understanding the language of life through self-supervised learning. *IEEE transactions on pattern analysis and machine intelligence*, 44(10):7112–7127, 2021.

Fang, Y. et al. DeepProSite: structure-aware protein binding site prediction using esmfold and pretrained language model. *Bioinformatics*, 39(12):btad718, 2023.

Jones, S. and Thornton, J. M. Analysis of protein-protein interaction sites using surface patches. *Journal of molecular biology*, 272(1):121–132, 1997.

Kahraman, A. et al. Shape variation in protein binding pockets and their ligands. *J molecular biology*, 368(1): 283–301, 2007.

Keskin, O. et al. Principles of protein- protein interactions: what are the preferred ways for proteins to interact? *Chemical reviews*, 108(4):1225–1244, 2008.

Kovtun, D. et al. PINDER: the protein interaction dataset and evaluation resource. biorxiv, 2024.

Kundrotas, P. J. et al. Dockground: a comprehensive data resource for modeling of protein complexes. *Protein Science*, 27(1):172–181, 2018.

Levy, E. D. A simple definition of structural regions in proteins and its use in analyzing interface evolution. *Journal of molecular biology*, 403(4):660–670, 2010.

Lin, Z. et al. Evolutionary-scale prediction of atomic-level protein structure with a language model. *Science*, 379 (6637):1123–1130, 2023.

Morehead, A. et al. DIPS-Plus: the enhanced database of interacting protein structures for interface prediction. *Scientific data*, 10(1):509, 2023.

Talavera, D., Robertson, D. L., and Lovell, S. C. Characterization of protein-protein interaction interfaces from a single species. *PloS one*, 6(6):e21053, 2011.

Touw, W. G. et al. A series of PDB-related databanks for everyday needs. *Nucleic acids research*, 43(D1):D364–D368, 2015.

Vreven, T. et al. Updates to the integrated protein–protein interaction benchmarks: docking benchmark version 5 and affinity benchmark version 2. *Journal of molecular biology*, 427(19):3031–3041, 2015.

Xue, L. C. et al. Protein-protein interface predictions by data-driven methods: a review. *FEBS letters*, 589(23): 3516, 2015.
